# A Study of Combined Onabotulinumtoxin A and Hyaluronic Acid Filler for the Treatment of Enlarged Facial Pores

**DOI:** 10.3390/toxins17010038

**Published:** 2025-01-15

**Authors:** Vasanop Vachiramon, Sonphet Chirasuthat, Suphagan Boonpethkaew, Nawara Sakpuwadol, Tanat Yongpisarn, Natthachat Jurairattanaporn

**Affiliations:** Division of Dermatology, Department of Medicine, Faculty of Medicine Ramathibodi Hospital, Mahidol University, Bangkok 10400, Thailand; vasanop.vai@mahidol.edu (V.V.);

**Keywords:** botulinum toxin, dermal filler, injectables, neuromodulators, pore size, skin quality

## Abstract

Introduction: Enlarged facial pores are a common cosmetic concern caused by excessive sebum production, visible hair shafts, and a reduction in skin elasticity, leading to a decrease in skin quality and overall appearance. Various treatment modalities have been explored to address this issue. This study focuses on the efficacy and safety of combining Onabotulinumtoxin A (OnaBoNT-A) and hyaluronic acid filler (HA filler) to target enlarged facial pores in Asians. Materials and Methods: This study aimed to compare the efficacy and safety of OnaBoNT-A monotherapy in combination with HA filler for the treatment of enlarged facial pores. This study was a prospective, randomized, single-blinded, split-face, controlled trial that enrolled 32 subjects with visibly enlarged pores on both cheeks. One side of the face received intradermal injections of OnaBoNT-A, while the other side received OnaBoNT-A in combination with intradermal hyaluronic acid filler injection. The outcomes were measured by pore volume, visual assessment, pain score, improvement score, and side effects at various time intervals up to 24 weeks. Results: This study investigated the effects of onaBoNT-A monotherapy or in combination with HA filler on facial pore size and skin roughness. The results showed that both sides exhibited a reduction in pore volume and skin roughness over time, but the side treated with onaBoNT-A monotherapy had a slightly better improvement than the combination side at the 6-month follow-up. Subjects with histories of facial oiliness were more likely to respond to onaBoNT-A monotherapy, while those without histories of facial oiliness were more likely to respond to the side treated with combined treatment. The most common adverse events were erythema, bruising, and edema, which were more frequent on the combination side. Additionally, 18 subjects (56.25%) experienced a palpable lump on the combination side, which resolved in most cases within a few months. Conclusion: BoNT-A and HA dermal filler had a role in reducing pore size. Nonetheless, individuals with enlarged pores who exhibited beneficial effects to botulinum toxin injection typically had a background of facial oiliness. Adverse incidents like dermal edema and palpable nodules were observed, underscoring the significance of meticulous patient selection and accurate injection technique.

## 1. Introduction

Enlarged facial pores are a common cosmetic concern for many individuals. Three major contributing factors include excessive sebum production, decreased elasticity around the pore area, and a thick, visible hair shaft [1]. Enlarged facial pores can also be exacerbated by external factors including sun exposure that leads to photoaging of the skin. Multiple treatment modalities can be used for the treatment of dilated pores in order to target the underlying causes of enlarged facial pores, such as topical vitamin A derivatives, lasers, energy-based devices, and botulinum toxin (BoNT) injection [2,3].

BoNT is a neurotoxin produced by the bacterium Clostridium botulinum that blocks the release of acetylcholine at the neuromuscular junction. BoNT was first used for therapeutic purposes in ophthalmology by Alan et al. in 1977, who injected BoNT type A (BoNT-A) into the extraocular muscles of monkeys to treat strabismus. Since then, BoNT has been widely adopted for various ophthalmologic conditions, such as blepharospasm, hemifacial spasm, and lagophthalmos [4,5,6]. BoNT has eight serotypes (A to H) that vary in potency and duration. BoNT-A and BoNT-B are used for therapeutic and cosmetic purposes, while the others are rare and less clinically relevant. Various BoNT-A that are commercially available include onabotulinumtoxin A (onaBoNT-A), abobotulinumtoxin A (aboBoNT-A), incobotulinumtoxin A (incoBoNT-A), leitibotulinumtoxin A (leiBoNT-A), prabotulinumtoxin A (praBoNT-A), and daxibotulinumtoxin A (daxiBoNT-A). They differ in manufacturing process, formulation, molecular weight, diffusion, and indications [7,8,9,10,11].

BoNT-A has been approved for cosmetic indications for decades including the treatment of glabella frown lines, forehead lines, and crow’s feet lines. Previous publications demonstrated that BoNT-A may have a potential role in the improvement of enlarged facial pores by targeting the underlying mechanisms that contribute to their dilation, including the reduction of sebum production and the stimulation of collagen around the pore areas [12,13,14,15,16].

Furthermore, hyaluronic acid (HA) filler is also known for its ability to augment volume and improve skin hydration around the treatment area. According to a recent study, the data have shown that HA dermal filler can help decrease the pore size by increasing the volume and also help stimulate new collagen synthesis around the pore area [17]. Moreover, the study of Vachiramon et al. demonstrated that HA filler injection in combination with microfocused ultrasound showed more improvement in pore size and better patient satisfaction compared to microfocused ultrasound alone [18].

Currently, there is a lack of research regarding the utilization of HA filler injection in combination with BoNT-A for the purpose of pore size reduction. The primary objective of this study is to determine the efficacy and safety of the combination of onabotulinumtoxin A (onaBoNT-A) and HA filler injection in decreasing enlarged facial pores among Asian patients.

## 2. Results

This study enrolled 32 healthy subjects with enlarged facial pores on both cheeks. The majority of subjects were female (87.5%) with a mean age of 35 ± 9 years. The most common Fitzpatrick skin type in this study was type III (50%), followed by type II and type IV (25% each). Regarding pre-existing comorbidities, there were no serious comorbidities reported from enrolled subjects. Baseline subject characteristics are demonstrated in Table 1.

### 2.1. Skin Roughness Measurement by Antera 3D^®^

**Figure 1:**
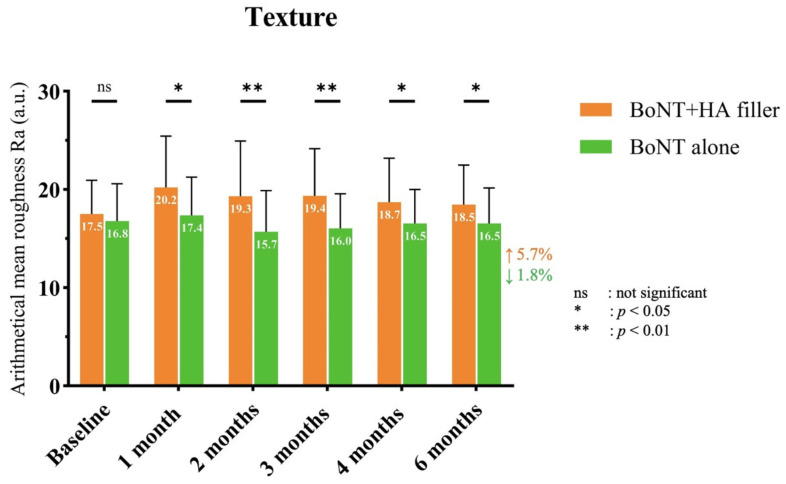
The comparison of skin roughness at baseline and follow-up visits between the combination of HA filler and onaBoNT-A and onaBoNT-A alone.

The Antera 3D^®^ skin roughness measurement data revealed that there was a similar level of skin roughness observed on both sides of the face at baseline (Figure 1). Upon reaching the 1-month follow-up visit, it was demonstrated that the side treated with the combination of onaBoNT-A and HA filler exhibited a notably higher average skin roughness in comparison to the side treated with onaBoNT-A monotherapy, and this variance was deemed statistically significant. Subsequent follow-up visits showed a decreasing trend in skin roughness for both the combination-treated side and the onaBoNT-A-treated side when compared to the baseline. Nevertheless, it was observed that the onaBoNT-A-treated side displayed a more pronounced enhancement in skin roughness than the combination-treated side, and this distinction was statistically significant at each follow-up visit.

### 2.2. Pore Volume Measurement by Antera 3D^®^

**Figure 2:**
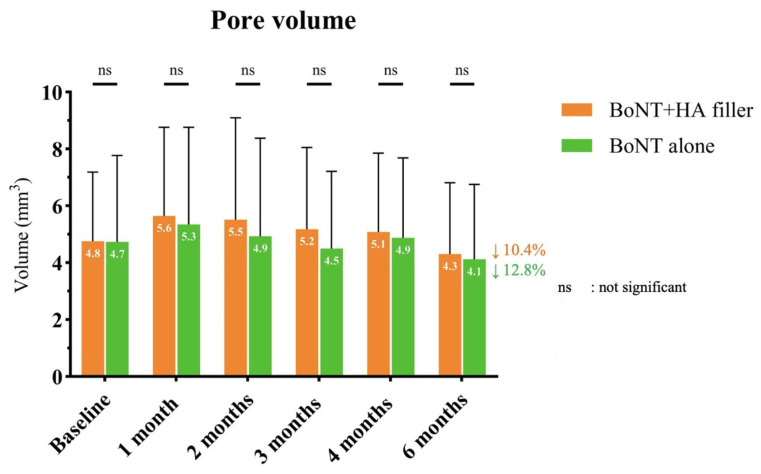
The comparison of pore volume at baseline and follow-up visits between the combination of HA filler and onaBoNT-A and onaBoNT-A monotherapy.

Regarding the measurement of pore volume using Antera 3D^®^, both sides exhibited similar pore volumes at baseline (Figure 2). During the 1-month follow-up, both sides of the facial area manifested an elevated pore volume, with a slightly higher level observed on the combination side, albeit without any statistically significant difference. Over subsequent follow-up visits, there was a noticeable trend towards pore size reduction on both sides of the face. Nevertheless, at the 6-month follow-up, the side treated with onaBoNT-A displayed a marginally superior enhancement in pore size in comparison to the baseline visit and outperformed the combination side during the same visit.

A subgroup analysis was conducted among individuals who exhibited a response and those who did not. There were 14 subjects out of the total 32 subjects (43.75%) who experienced a minimum of 1-grade improvement in pore size on the side that was treated with onaBoNT-A monotherapy. A notable observation was the higher occurrence of a history of oiliness among these subjects in comparison to the non-responders (78.57% versus 22.2%, *p* < 0.05) as shown in Table 2.

Additional analysis focused on the improvement in pore size following the administration of a combination of HA filler and onaBoNT-A. The findings indicate that individuals who showed favorable results to the combination treatment (6 out of 32 participants) did not have a prior record of facial oiliness in contrast to the non-responders (16.67% versus 53.84%, *p* < 0.05) as shown in Table 3.

### 2.3. Visual Assessment Criteria for Pores Evaluated by Physician

**Figure 3:**
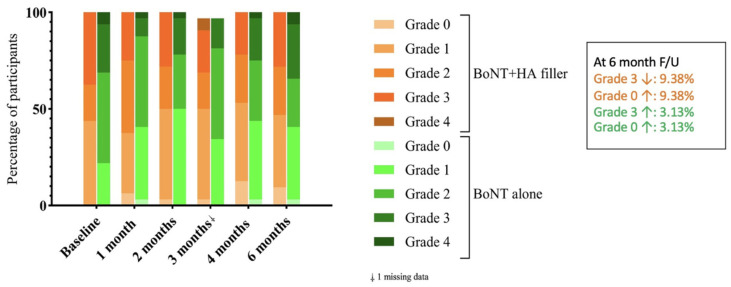
The visual assessment criteria for pores evaluated by the physician compared the combined HA filler and onaBoNT-A vs. onaBoNT-A monotherapy.

The visual assessment criteria for pores, evaluated by the physician, focus on identifying any changes in pore appearance at each follow-up visit, graded from 0 to 4, from less to most visible pore (Figure 3). There was an increasing proportion of subjects that had better improvement grading of pore size on both sides of the face. However, at the last follow-up visit, there was an increased proportion of patients with grade 0 (less visible) pores on the combination side of 9.38%, while on the onaBoNT-A side, it was 3.13%.

### 2.4. Improvement Score Evaluated by Subjects

**Figure 4:**
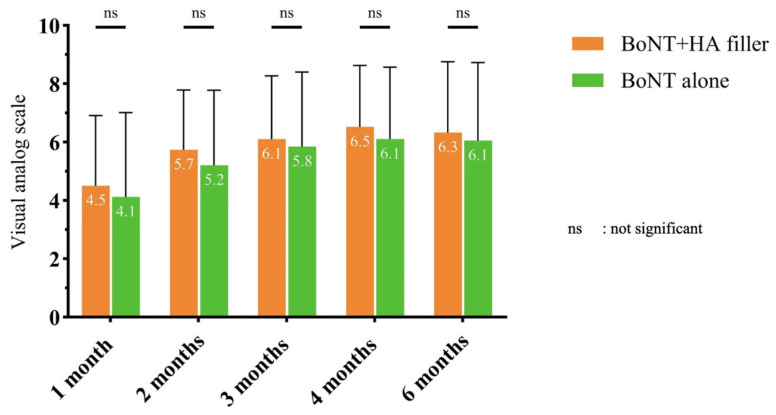
The improvement scores rated by subjects between the combined side and the onaBoNT-A side at baseline and follow-up visits.

**Figure 5:**
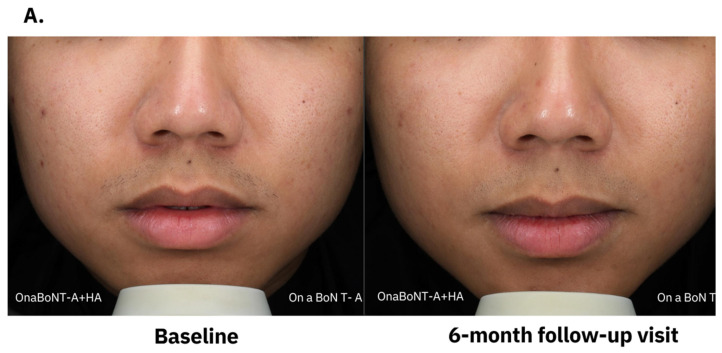

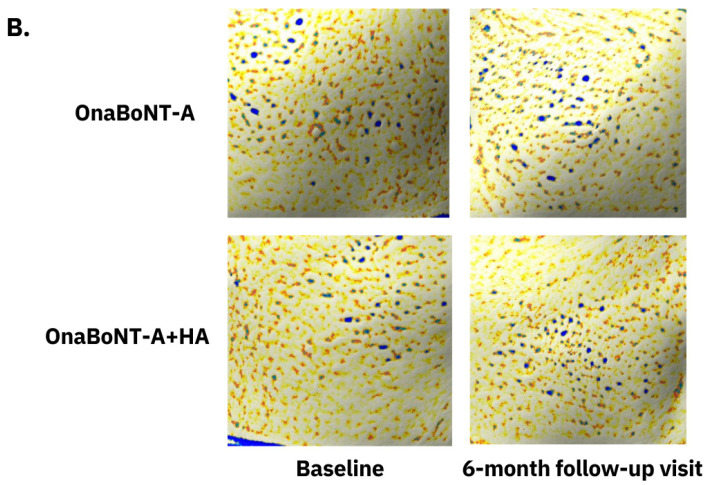
The clinical photographs (**A**) and pore size detection images from Antera3D^®^ (**B**) of representative subject at baseline and 6-month follow-up visit.

The study subjects assessed the improvement score utilizing a visual analog scale ranging from 0 to 10, both at the baseline and 1, 2, 3, 4, and 6 months postintervention (Figure 4). Findings indicated that the combination of HA filler with onaBoNT-A produced superior improvement scores in contrast with the use of onaBoNT-A alone. Nevertheless, no statistically significant difference was observed during each follow-up visit. The clinical outcomes from both photographs and the Antera3D^®^ system between the initial baseline assessment and the subsequent follow-up examination conducted after a period of six months are demonstrated in Figure 5.

### 2.5. Adverse Events

Subjects were asked to rate the pain score during the procedure using a visual analog scale ranging from 1 to 10, and the results showed that most of the subjects reported minimal and tolerable discomfort. The average pain score was found to be 3.3 ± 2.4 on the combination side and 3.4 ± 2.3 on the side where onaBoNT-A was used as a monotherapy. There was no statistically significant difference between the two sides in terms of pain score (*p* = 0.65). Bruising was observed in eight patients (25%) and 15 patients (46.88%) in the BoNT-A monotherapy and combined groups, respectively.

**Figure 6:**
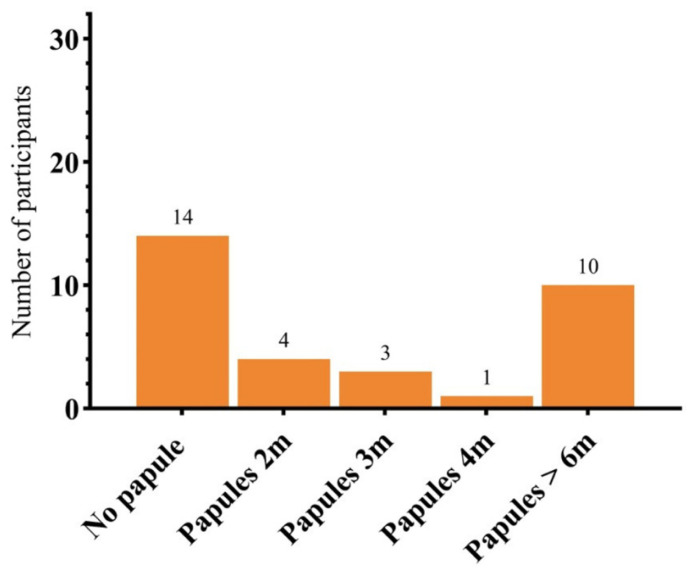
The occurrence of papule(s) on the combined side at each follow-up visit.

At subsequent follow-up visits, 18 subjects (55.56%) experienced a dermal papule on the side that had been treated with a combination of HA filler and onaBoNT-A at a 1-month follow-up. Ten subjects had a persistent papule(s) for up to 6 months, while the rest of the subjects reported that the papules resolved after a few months (Figure 6).

## 3. Discussion

Enlarged facial pores are a common cosmetic concern that affects the appearance and quality of the skin. Pore size is influenced by several factors, such as excessive sebum production, visible hair shafts, and a reduction in skin elasticity [1]. Management can be targeted by various treatments depending on the etiological factors contributing to the appearance of pore size, such as topical agents, oral medications, lasers, and energy-based devices [19,20,21,22,23].

Cosmetic injectables, including BoNT-A and HA filler, are gaining more popularity nowadays for applications in wrinkle reduction and volume enhancement, respectively [1]. BoNT-A is a neurotoxin that blocks the release of acetylcholine from the presynaptic nerve terminals, resulting in temporary muscle paralysis. It has been widely used for cosmetic purposes, such as reducing facial dynamic wrinkles and reshaping the face [24,25]. It has also been widely used for the management of hyperhidrosis [26]. Recently, botulinum toxin has also been shown to reduce sebum production and pore size by direct effects on sebocytes, reducing oleic acid-induced lipogenesis, potentially offering treatment for sebum-related conditions like oily skin and acne [14,27,28]. Recent publications also demonstrated that BoNT-A effectively improved sebum secretion, face laxity, and facial pores. Results showed significant improvements after 4 weeks of treatment using microdroplet injections. All subjects reported better outcomes on the Global Aesthetic Improvement Scale, indicating its effectiveness for face lifting and pore reduction [13]. In this study, pore size reduction was observed in 43.75% of subjects with BoNT-A monotherapy. This group of patients had a previous history of facial oiliness that may indicate active sebaceous gland function. This can serve as a target for BoNT-A and result in the improvement of pore size.

For HA filler, it has been widely used to restore volume and contour including midface volume loss, infraorbital hollowness, temporal augmentation, nasolabial folds improvement, lips enhancement, etc. Recently, HA filler has also been used for skin rejuvenation and texture improvement by injecting small amounts of low-crosslinked HA into the dermis. According to previous studies, the injection of intradermal low-crosslinked HA demonstrated an improvement in pore size [17,18]. VYC-12L (Juvéderm Volite; Allergan plc, Dublin, Ireland) is a cross-linked HA filler that is designed for intradermal injection with a low concentration (12 mg/mL) and a high cross-linking degree. Throughout a series of various appointments for the VYC-12L procedure, which comprises the primary treatment session, a follow-up visit for touch-ups, and another treatment during the 9-month check-up, research has proven that significant enhancements in skin hydration, elasticity, and roughness are observed. These improvements have been demonstrated to be long-lasting, persisting for a period of up to 10 months after the first visit for the procedure [29]. Moreover, the histological assessment of the dermal layer of the volar forearm, which received intradermal injections of VYC-12 L, revealed elevated levels of epidermal aquaporin-3 (AQP3) and Ki67 expression at both 1 and 3 months following the treatment. Additionally, there was a noticeable increase in the presence of collagen type I in the papillary dermis at the 3-month duration [30].

The combination of BoNT-A and HA filler may theoretically have synergistic effects on pore size reduction and skin texture improvement by targeting different mechanisms. BoNT-A can reduce sebum production and pore size by inhibiting the pilosebaceous unit, while HA filler can hydrate and smooth the skin by filling the dermal space and stimulating collagen synthesis. The results of our study have shown that the combination treatment of BoNT-A and HA filler injection for enlarged pores showed more improvement on the combined side based on patients’ VAS, but there was no significant difference when compared to BoNT-A monotherapy. In addition, persistent dermal papule(s) were present in 55.56% of the subjects at 1-month follow-up. This can be caused by the excessive injection of HA filler in a relatively small area (i.e., 1 mL of filler into the pore area of one side of the face). In addition, the depth of injection in this study was the superficial dermis. Based on a previous study, The recommended injection technique of VYC-12L includes the injection into the deep dermis with a 32G ½ inch needle inserted at <45° to the skin, spaced 0.5–1.0 cm apart, with 0.01–0.05 mL volume per injection [12].

Hence, it is crucial to carefully consider the selection of suitable cases as well as the injection technique as these factors play a significant role in enhancing the effectiveness of the combined therapy. Individuals who possess oily skin with noticeable excess sebum production might experience greater advantages from the utilization of BoNT-A, whereas individuals with dry skin, subtle fine lines, and loss of elasticity around pore areas may find greater benefits from the use of HAfiller. Furthermore, the technique used for injecting this particular type of HA filler involves carefully determining the appropriate quantity to be used for each specific area of the face. The injection should be performed at a proper depth to achieve the desired outcome of creating a less edematous appearance of the skin.

## 4. Conclusions

The use of BoNT-A for enlarged facial pores showed a promising result in patients with a history of facial oiliness, whereas patients with favorable responses to additional HA filler were less likely to have histories of facial oiliness. Proper patient selection along with the appropriate amount and technique for each injectable product are important for achieving a desirable cosmetic outcome.

## 5. Materials and Methods

This study was designed as a prospective, randomized, single-blinded, split-face, controlled study conducted at Ramathibodi International Training Center for Laser Therapy, Ramathibodi Hospital, Mahidol University, Bangkok, Thailand. This study was conducted in accordance with the Declaration of Helsinki and approved by the Institutional Review Board of the Faculty of Medicine, Ramathibodi Hospital, Mahidol University (Protocol number: MURA2023/90 Ref. 3762). This study was registered to Thai Clinical Trials Registry (TCTR20250115002). The participants included 32 subjects who presented with enlarged facial pores on both cheeks.

### 5.1. Study Subjects

This study included subjects aged 18 to 65 years with visibly enlarged pores on both sides of the face. Exclusion criteria encompassed the following: a known history of allergy to botulinum toxin or HA filler, a history of neurological disease or deficit, concurrent severe underlying or uncontrolled diseases such as autoimmune connective tissue diseases, a history of systemic retinoids intake within 3 months, a history of lasers or energy-based devices treatment on both cheeks within 3 months, and the presence of skin infection or active acne in the treated area. The participants were recruited from an outpatient dermatology clinic and were required to provide informed consent before being enrolled in this study. Baseline demographic data were recorded, including age, sex, underlying comorbidities, Fitzpatrick’s skin type, and history of facial oiliness. The subjects demonstrating elevated levels of sebum secretion are those who exhibit a rapid resurgence in sebum production within a time frame of 3 h following facial cleansing [31].

### 5.2. Randomization and Blinding

A block randomization method was conducted to allocate patients into the following two sides: one side receiving onabotulinumtoxin A (onaBoNT-A) and the contralateral side receiving onaBoNT-A in combination with same-session HA filler injection. The injections of both onaBoNT-A and HA filler were administered by a dermatologist.

### 5.3. Treatment Protocol

**Figure 7:**
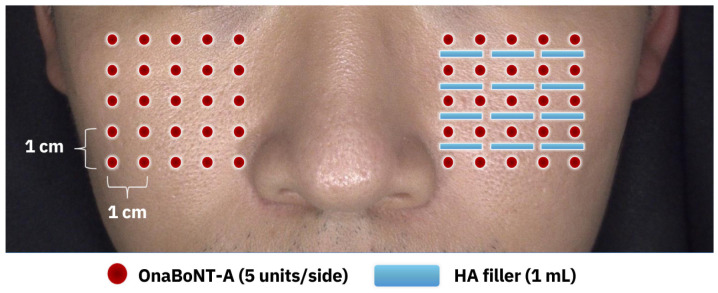
Represents injection point of onaBoNT-A and HA filler on both cheeks.

Regarding the preparation of neurotoxin, onaBoNT-A (BOTOX^®^; Allergan Aesthetics, an AbbVie Company, Irvine, CA, USA) was diluted with 2 mL of 0.9% NaCl in a 100-unit bottle. Five units of onaBoNT-A was further diluted with normal saline to 1 mL and injected into the superficial dermis in a microdroplet fashion on an enlarged pore area on a single side of the face. The contralateral side was also injected with an identical dosage and depth of onaBoNT-A solution. The HA filler used in the combination side was cross-linked HA gel (Juvéderm^®^ Volite; Allergan Aesthetics, an AbbVie company, Irvine, CA, USA). The injection technique involved a linear retrograde threading of HA filler in the superficial dermis with a 30-degree angle of needle insertion on the enlarged pore area with a total amount of 1 mL on the combination side. The injection plan of both onaBoNT-A and HA filler is demonstrated as shown in Figure 7.

### 5.4. Outcome Measurement

The objective evaluation is performed by an experienced dermatologist through the analysis of photographs taken using Antera 3D^®^ (Miravex Limited, Dublin, Ireland) pre- and post-treatment at 4, 8, 12, 16, and 24-week intervals. Pore volume is determined using a medium filter from 3D images of both cheeks and expressed as continuous ^#-in cubic millimeters (mm^3^) on each side of the face.

For the subjective evaluation by a physician, utilizing VISIA^®^ (Canfield Scientific, Parsippany, NJ, USA) images taken before and after treatment at specific intervals, measurements are taken from the largest pores in a selected square area of 2 × 2 cm^2^, 300 pixels, and assessed by a blinded dermatologist using visual assessment criteria for pores on a scale of 0–4 for each side of the face. Moreover, the subjective evaluation by subjects includes pain scores and improvement scores. Pain levels are evaluated immediately following treatment using the Visual analog scale on both sides of the face. The improvement in pore condition post-treatment at specified intervals compared to pre-treatment images captured by VISIA^®^ is assessed using the Visual analog scale (VAS) on both sides of the face.

**Figure 8:**
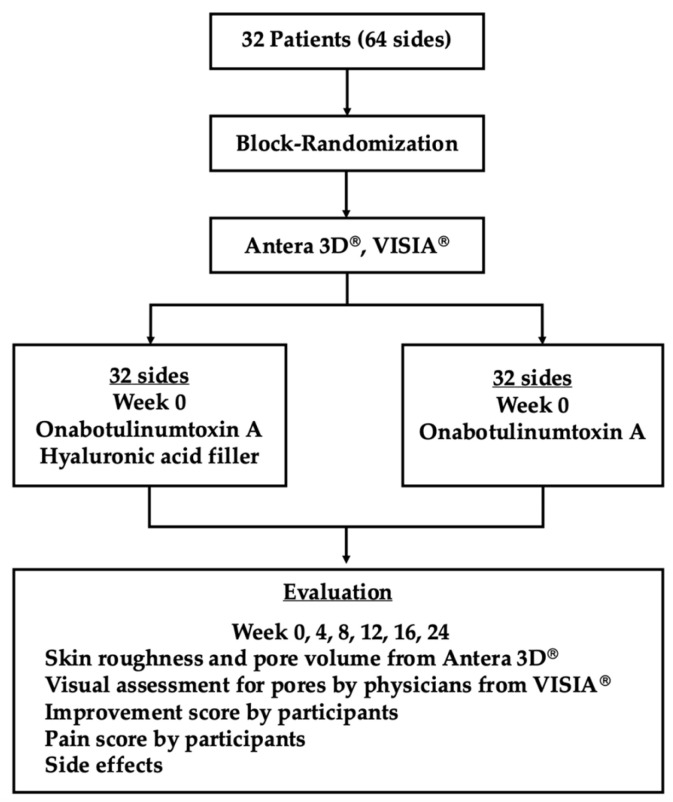
Demonstrates the flowchart of the study protocol.

The side effects are evaluated by a dermatologist, encompassing redness, swelling, skin peeling, and scarring post-treatment, with evaluations taking place immediately post-treatment and at subsequent follow-up visits for each side of the face. The study subjects also evaluate side effects, including pain, burning sensation, redness, swelling, and symptom duration post-treatment and at follow-up visits, on each side of the face. The flowchart of the study is demonstrated in Figure 8. 

### 5.5. Statistical Analyses

Descriptive statistics included means and standard deviations (SD) for continuous variables and percentages for categorical variables. The primary outcome was the comparison between the combination of onaBoNT-A with HA-filler and the onaBoNT-A-treated side. The analysis of pore volume and participant satisfaction was conducted using a linear mixed-effect model, while the analysis of pore severity scores was performed using a multilevel mixed-effect ordered logistic regression. All statistical analyses were conducted using STATA version 17.0 (StataCorp^®^. 2021. Stata Statistical Software: Release 17. StataCorp LCC, College Station, TX, USA). The threshold for statistical significance was set as a *p*-value of less than 0.05 (two-sided).

## Figures and Tables

**Table 1:** Baseline subject characteristics.

Characteristics	*n* (%)
Sex (*n*, %)	
Male	4 (12.50)
Female	28 (87.50)
Age (year, Mean ± SD)	35.0 ± 9.0
History of facial oiliness (*n*, %)	15 (46.88)
Fitzpatrick skin type (*n*, %)	
Type II	8 (25.00)
Type III	16 (50.00)
Type IV	8 (25.00)
Comorbidity (*n*, %)	
Allergic rhinitis	2 (6.25)
Hypertension	1 (3.13)
Dyslipidemia	1 (3.13)
Hypothyroidism	1 (3.13)

Abbreviation: SD, standard deviation.

**Table 2:** Data between responders and non-responders to onaBoNT-A monotherapy.

Characteristics	Responders (*n* = 14)	Non-Responders (*n* = 18)	*p* Value
Age (mean ± SD)	34.0 ± 8.3	35.8 ± 10.0	0.5014 *
Male gender, *n* (%)	1 (7.14%)	3 (16.67%)	0.6128 **
History of facial oiliness, *n* (%)	11 (78.57%)	4 (22.22%)	<0.05 **
Fitzpatrick skin type, *n* (%)			
Type II	4 (28.57)	4 (22.22%)	0.7035 **
Type III	7 (50.00%)	9 (50.00%)	>0.9999 ***
Type IV	3 (21.43%)	5 (27.78%)	>0.9999 **

* Unpaired *t*-test, ** Fisher’s exact test, *** Chi square test.

**Table 3:** Data between responders and non-responders to combined onaBoNT-A and HA filler therapy.

Characteristics	Responders (*n* = 6)	Non-Responders (*n* = 26)	*p* Value
Age (mean ± SD)	36.3 ± 9.5	34.7 ± 9.3	0.8288 *
Male gender, *n* (%)	0 (0.00%)	4 (15.38%)	0.5662 **
History of facial oiliness, *n* (%)	1 (16.67%)	14 (53.84%)	<0.05 **
Fitzpatrick skin type, *n* (%)			
Type II	1 (16.67%)	7 (26.92%)	>0.9999 **
Type III	4 (66.67%)	12 (46.15%)	0.6539 **
Type IV	1 (16.67%)	7 (26.92%)	>0.9999 **

* Unpaired *t*-test, ** Fisher’s exact test.

## Data Availability

The original contributions presented in this study are included in this article. Further inquiries can be directed to the corresponding author.
